# Editorial to the Special Issue “Information Processing in Neuronal Circuits and Systems”

**DOI:** 10.3390/biology12030359

**Published:** 2023-02-24

**Authors:** Alireza Valizadeh, Claudio Mirasso

**Affiliations:** 1 Department of Physics, Institute for Advanced Studies in Basic Sciences (IASBS), Zanjan 4513766731, Iran; 2 Pasargad Institute for Advanced Innovative Solutions (PIAIS), Tehran 1991633357, Iran; 3 Instituto de Física Interdisciplinar y Sistemas Complejos, IFISC (UIB-CSIC), Campus Universitat de les Illes Balears, E-07122 Palma de Mallorca, Spain

The nervous system processes sensory information through a hierarchical structure with multiple processing stages. The convergent-divergent connectivity pattern has been shown to efficiently convey information about external stimuli. In addition, there are abundant downstream connections, as well as horizontal connections within and between layers, which facilitate high-level processing and modulate the response of the nervous system to external stimuli, both as a function of the temporal and spatial context and the current state of the system [1].

Proper brain function relies on the integration of information processed in local circuits, and it is a key question to understand how the brain integrates the multitude of local processes throughout its anatomy. Communication between brain regions involves the transfer of electrical activity coded in different local neural populations, which is quantified by statistical relationships between brain areas derived from brain imaging or electrophysiological recordings. This communication, known as "functional connectivity" [2,3]. The experimental results show that functional connectivity is dynamic, highlighting that the pattern of the communication between different brain regions and the routes for information exchange are subject to continuous and rapid changes due to environmental conditions or the state of the nervous system. 

The flexibility of information routing and effective connectivity stems from the multistability of neuronal network dynamics. This means that a single structural connectivity pattern can result in a variety of dynamic states, each with a unique pattern of communication. Collective neuronal oscillations are believed to play a role in dynamic communication between brain regions by modulating the exchange of phase information between connected areas through adjustments in the phase relations. [4,5]. 

This Special Issue delves into the intricacies of communication and information processing within neuronal circuits and systems. The articles explore how various structural organizations of brain circuits can impact their dynamics and functionality, as well as the role of brain oscillations in regulating sensory processing, motor functions, and inter-brain communication. The following sections provide a comprehensive overview of the papers published in this Special Issue.

How information propagates during sleep–waking cycles was tackled by Farhad Razi and co-workers [6]. In this study, the hypothesis that during sleep the responses of the primary auditory cortex to sounds are weakened through thalamic gating was challenged. Instead, it was found that during non-rapid eye movement (NREM) sleep, cortical responses to sounds remain local and only reach nearby neuronal populations. The researchers used a computational neural-mass model to understand how this behavior emerges as the brain shifts between NREM sleep and wakefulness. The study found that an increase in the excitatory conductance in the model can place it from NREM sleep to wakefulness, producing a similar or higher response to excitatory inputs. The researchers also found that it is the inter- and intra-conductance ratio of cortical excitatory synapses, rather than an increase in all the conductances, that needs to be raised to facilitate information propagation across the brain.

A model of the early visual system was developed by Alejandro Santos-Mayo and co-workers [7]. In this work, the authors present a reduced visual system model (RVSM) of the first level of scene analysis, involving the retina, the lateral geniculate nucleus, and the primary visual cortex (V1). The model is based on the neuromorphic spike-decoding structure, which is able to learn and recognize parallel spike sequences. This structure is equipped with the plasticity of intrinsic excitability to embed recent findings about V1 operation. The RVSM is tested with sets of rotated Gabor patches as the input and the synthetic visual evoked activity generated by the model is compared to the real neurophysiological signal from the V1 area. The results attest to a good level of resemblance between the model response and real neurophysiological recordings.

The mechanisms of flexible information sharing supported by noisy oscillations was studied by Powanwe and Longtin [8]. The authors study the relationship between the phase synchronization and information sharing in two coupled oscillatory networks. The networks are modeled as excitatory–inhibitory nodes connected through zero-delay excitatory connections. The phase-locking and delayed mutual information between the phases of the excitatory local field potentials of the two networks are calculated to measure the shared information and its direction. The results show that the flexibility in information sharing depends on the origin of the oscillations, with noise-perturbed rhythms exhibiting two local information maxima, while coupled noise-induced rhythms do not show flexibility. The mechanisms reported can be extended to inhibitory–inhibitory networks. The study provides insights into information sharing in complex biological networks.

The optimal input representation in neural systems operating at the edge of chaos was investigated by Morales and Muñoz [9]. The authors explore the idea that operating at the “edge of chaos” or criticality can provide advantages for information processing in biological systems. They study the statistics of the covariance matrices of a neural network trained to classify images and find that the best performance is achieved when the network operates near the critical point, with the eigenspectrum of the covariance matrix following the same statistics as observed in neurons of the mouse visual cortex. The results suggest that operating near criticality allows for flexible, robust, and efficient input representations.

The functional interaction between the entorhinal cortex input and theta activity in the hippocampus was studied by López-Madrona and Canals [10]. The authors study the information transmission between multiple theta rhythms in the hippocampus during context exploration and memory formation. They use source separation techniques and analytical tools to investigate directed interactions between the theta activities recorded in rats exploring a known environment with or without a novel stimulus. The results show that exploration in the novelty condition is associated with increased theta power in the generators with an entorhinal cortex origin and increased directed functional connectivity from the entorhinal cortex layer II to III.

Post-scratch locomotion in cats was studied by Tapia and co-workers [11]. The study presents a model of two central pattern generator (CPG) networks for post-scratching locomotion. The model is based on the hypothesis that the rhythm generator layers for scratching and locomotion are different but share supraspinal circuits and motor outputs. The model successfully reproduces observed post-scratching locomotion latency and cycle durations from a previous experimental study. The findings suggest that an integrated organization of two rhythmic movements may provide flexible and effective connectivity through a common pattern formation layer.

The relation between EEG and synaptic fickleness was studied by Pretel and co-workers [12]. This study investigates a network of excitatory and inhibitory neurons that produces oscillations similar to brain waves. The findings show that changes in the EEG series are due to restrictions in the synaptic activity causing an imbalance between excitation and inhibition. This leads to a transition in the dynamic mental phases, and the model exhibits delta–theta and fast oscillations. These findings offer a new way of understanding brain data.

The self-organized structuring of recurrent neuronal networks was considered by Miner and co-workers [13]. The study explores how the brain processes information using a layered hierarchical network architecture. It shows that the cortex can self-organize in response to new inputs by a well-orchestrated interplay of plasticity processes. This allows for rapid information routing between sparse input and output connections by forming feed-forward projections within the same layer. The plasticity processes ensures that each neuron only responds to one stimulus, dividing the network into parts with different preferred stimuli. The resulting network activity and connectivity minimize the metabolic cost for transmitting information efficiently.

Distinct locomotor behaviors in Drosophila larva were studied by Gowda and co-workers [14]. The authors focus on the study of movements in Drosophila larvae and the underlying neural circuitries and pathways involved. Despite the complexity of movement generation and sensory information integration, larvae provide a genetic tractable model to study the behavior response to sensory modalities. The review highlights the role of interneurons in regulating normal and defensive movements, providing an insight into the genetic and physiological components of movement. The aim is to better understand the mechanisms of movement and how different locomotion types are achieved.
